# Ce^3+^ Doped Al_2_O_3_-YAG Eutectic as an Efficient Light Converter for White LEDs

**DOI:** 10.3390/ma16072701

**Published:** 2023-03-28

**Authors:** Anna Shakhno, Tetiana Zorenko, Sandra Witkiewicz-Łukaszek, Mieczysław Cieszko, Zbigniew Szczepański, Oleh Vovk, Sergii Nizhankovskyi, Yuriy Siryk, Yuriy Zorenko

**Affiliations:** 1 Department of Physics, Kazimierz Wielki University in Bydgoszcz, 85090 Bydgoszcz, Poland; 2 Mechantronic Department, Kazimierz Wielki University in Bydgoszcz, 85074 Bydgoszcz, Poland; 3 Institute for Single Crystals, National Academy of Sciences of Ukraine, 61178 Kharkiv, Ukraine

**Keywords:** Al_2_O_3_-Y_3_Al_5_O_12_ eutectic, Ce^3+^ dopant, luminescence, phosphor converters, white LEDs

## Abstract

Ce^3+^ doped Al_2_O_3_-YAG eutectics were successfully grown by the horizontal directional crystallization method. The crystallization rate of eutectic growth was changed in the 1–7.5 mm/h range at a growth temperature of 1835 ℃. The microstructure of eutectic samples was investigated using scanning electron microscopy and X-ray microtomography. The intrinsic morphology of eutectic represents the stripe-like channel structure with a random distribution of the garnet Y_3_Al_5_O_12_ (YAG) and Al_2_O_3_ (sapphire) phases. The content of these phases in the stripes changes in the 52.9–55.3% and 46.1–47.1% ratios, respectively, depending on the growth rate of the crystallization of the eutectic samples. The luminescent properties of the eutectic demonstrated the dominant Ce^3+^ luminescence in the garnet phase. The luminescence of the Ce^3+^ ions in Al_2_O_3_ has also been observed and the effective energy transfer processes between Ce^3+^ ions in the Al_2_O_3_ and YAG garnet phases were revealed under high-energy excitation and excitation in the UV Ce^3+^ absorption bands of sapphire. The phosphor conversion properties and the color characteristics (Al_2_O_3_-YAG):Ce eutectic with different thicknesses were investigated under excitation by a blue LED. We have also tested the prototypes of white LEDs, prepared using a blue 450 nm LED chip and (Al_2_O_3_-YAG):Ce eutectic photoconverters with 0.15 to 1 mm thicknesses. The results of the tests are promising and can be used for the creation of photoconverters for high-power white LEDs.

## 1. Introduction

One of the most promising areas of artificial lighting sources is connected to the production of white light-emitting diodes (WLEDs) due to their remarkable properties, such as a long lifespan, high efficiency, compactness, thermal stability, environmental friendliness, and low voltage operation over conventional light sources. The production of the current WLED lighting system is an established technology that typically combines a blue chip (GaN and InGaN) with a yellow-emitting Y_3_Al_5_O_12_:Ce (YAG:Ce) powder phosphor converter (pc) dispersed in silica gel or epoxide resin binder, which are used for fixation powder phosphors onto blue LED chips [1,2,3,4,5]. However, the mentioned organic binders with limited thermal conductivity and poor thermal stability are not suitable for high-power WLEDs. This brings up some problems in the actual use of WLED, such as deterioration of the light efficiency, chromaticity shift, and, eventually, the loss of long-term reliability.

Novel durable phosphors without resin binders have been studied by researchers to address the issue [6,7,8]. Completely inorganic phosphors, such as transparent ceramic phosphor and glass ceramic phosphor [9,10], are on track to displace the “phosphor powder” and “organic matrix” combination as workable solutions to the degradation of the organic resin matrix. Researchers have explored innovative durable phosphors without resins [6,11] and found the need to increase the phosphor scattering [12] to solve these problems. In this frame, the composites of the (Al_2_O_3_-YAG):Ce eutectic attract large interest as an excellent matrix of the phosphor converters due to their higher thermal conductivity in comparison with the YAG:Ce converter [13].

In addition to the above, one of the primary criteria that characterizes low light converter efficiency is the impact of total inner reflection, which transforms a light converter with a regular shape into a waveguide that traps a significant amount of light rays inside itself. This effect can be reduced using a variety of techniques, including adding scattering metal-oxide particles of micron sizes to the converter material [14,15], profiling the light converter’s emitting surface [16,17], and using a eutectic compound made up of multiple phase components [10,18,19,20,21]. It has been shown in these works that their lighting properties (Al_2_O_3_-YAG):Ce eutectic were significantly improved compared to traditional materials. In addition, due to the thermal stability of the Ce^3+^ emission, heavy Ce^3+^ doped YAG-Al_2_O_3_ eutectic has been considered as one of the prospective inorganic materials for pc for high-power warm WLEDs [10,14].

In this work, we have investigated the properties of highly doped Ce^3+^ doped Al_2_O_3_-YAG eutectic samples, which were crystalized by the horizontal directional crystallization (HDC) method [22] with different crystallization rates. Namely, the current work presents the new systematic results of studying the morphology, structural, and luminescent properties of these materials. Furthermore, the color characteristics and phosphor conversion capabilities of WLED prototypes with pc based on the (Al_2_O_3_-YAG):Ce eutectic samples with various thicknesses (from 0.15 to 1 mm) were investigated.

## 2. Sample Preparation and Experimental Techniques

Crystallization of Ce-doped Al_2_O_3_-YAG eutectic samples was carried out using the HDC method on the “Horizont-3M” set up in a Mo crucible under an Ar, CO, and H_2_ environmental atmosphere at a total pressure of 1.3 × 10^5^ Pa at evaluated temperature and rate of solidification.

**Figure 1:**
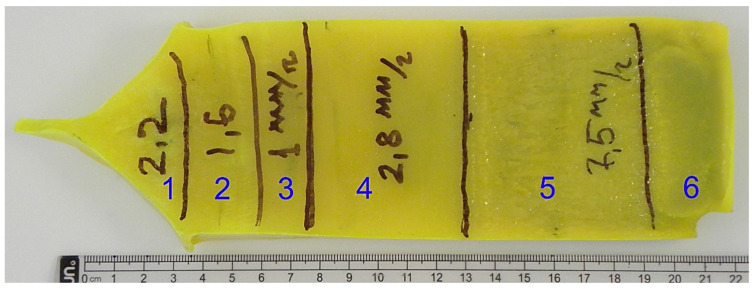
The (Al_2_O_3_-YAG):Ce eutectic ingot, different parts of the ingot grown at different crystallization rates (Table 1).

For the preparation of the initial eutectic charge, an equimolar mixture of Y_2_O_3_ + CeO_2_ (5N purity) and Al_2_O_3_ (4N purity) oxides was used. These powders were thoroughly mixed in a drum for several hours and the resulting mixture was tableted. The tablets were annealed at 1200 °C for several hours and the annealed tablets served as a “green body” for crystallization. Crystallization was performed by the HDC method on standard installations for Mo crucibles with a size of 240 × 75 × 35 mm^3^, according to the modes for the YAG crystals’ growing method [22]. During crystallization, different parts of the crystal were grown with different crystallization rates (Figure 1 and Table 1).

The nominal CeO_2_ content in the melt was 1 molar %. Moreover, due to the low segregation coefficient of Ce^3+^ ions, the actual Ce content in the Al_2_O_3_ and YAG phases can be notably less. For instance, the cerium segregation coefficient at the growth of YAG:Ce crystals is 0.2–0.25 [23]. However, we cannot measure the actual Ce^3+^ content in the garnet and sapphire phases separately. Carbon dopant may also be present in small amounts in the eutectic samples due to the carbon lining of the growth chamber being used.

The crystallized (Al_2_O_3_-YAG):Ce eutectic ingot and parts of the different crystallization rates are demonstrated in Figure 1. The surface of the eutectic crystal is marked in Section 1, Section 2, Section 3, Section 4, Section 5 and Section 6 which was crystallized at different growth rates (Table 1). Samples from Section 2, Section 3, Section 4 and Section 5 were investigated in this work. Samples were sliced forward perpendicular in the direction of crystal growth. There were no inclusions or cracks visibly seen in the eutectic slices, which had the bright yellow hue typical of Ce-doped YAG crystals.

The cooled crystal was cut into pieces that were grown at the same rate. Samples for research were then cut out of each part. Cutting was performed with diamond circular saws using water for cooling. The surfaces of the samples were polished with a diamond abrasive, and the samples were glued to the polishing table with paraffin wax. This wax was then wiped with a solvent.

The structural properties of these samples were characterized by electronic scanning microscopy (SEM, JSM-6390LV, JEOL Ltd., Tokyo, Japan), X-ray diffractions (modified DRON 4 spectrometer), and X-ray microtomography with a 0.5 µm resolution (SkyScan 1272 spectrometer). Namely, Table 1 shows the data obtained on the basis of analyses of the SEM surface images. In this instance, image analysis with the use of special software, installed in our SEM, allowed us to examine SEM images and determine the area fractions of various phases, which were distinguished by their contrast or color. The software can be calibrated to the specific magnification and pixel resolution of the images to ensure respective accuracy.

For characterization of the properties of the Ce^3+^ doped Al_2_O_3_-YAG eutectic samples under study, scanning electron microscopy (SEM), X-ray diffractions (XRD), X-ray microtomography (µCT), cathodoluminescence (CL), photoluminescence (PL), PL excitation spectra (PLE), PL decay kinetics, and photoconversion spectra (PC) were used. Furthermore, the photoconversion properties of the (Al_2_O_3_-YAG):Ce eutectic samples (color chromaticity coordinates (CIE), color correlated temperature (CTT), and color rendering index (CRI)) and luminous efficacy (LE) under blue LED excitation were investigated as well.

CL spectra were obtained by using an electron gun of a SEM JEOL JSM-820 microscope (JEOL Ltd., Tokyo, Japan), additionally equipped with a Stellar Net spectrometer with a cooled TE-detector CCD operating in the 200–1200 nm range. PL emission and excitation spectra, as well as PL decay kinetics, were measured using an FS-5 spectrometer (Edinburg Instruments Ltd., Livingston, UK). The photoconversion spectra (PC) measurements were performed using a fiber-optic spectrophotometer, AvaSpec-ULS 2048-LTEC, and an integrating sphere, AvaSphere-50-IRRAD. The photoconverters prepared from (Al_2_O_3_-YAG):Ce eutectics were excited by the blue LED (30 mA, 2.9V) with a wavelength of 454 nm. All measurements were performed at room temperature (RT).

## 3. Structural Properties of Eutectics

**Figure 2:**
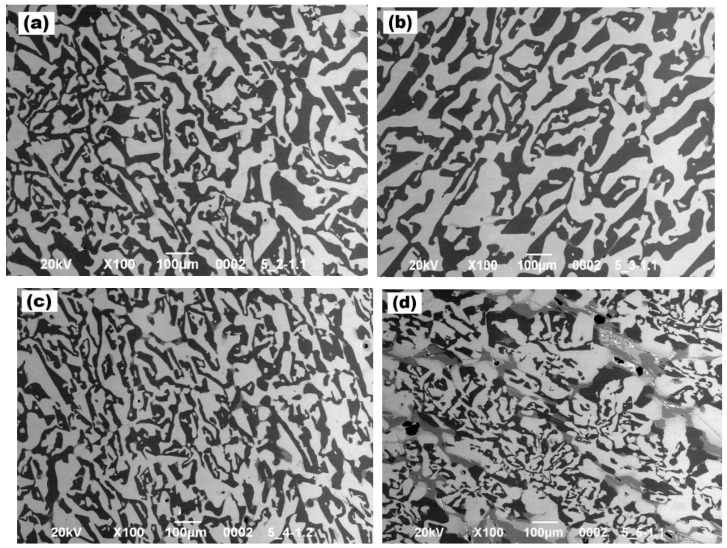
SEM images of the (Al_2_O_3_-YAG):Ce eutectic: (**a**) sample 2, (**b**) sample 3, (**c**) sample 4, and (**d**) sample 5.

SEM images of the morphology of (Al_2_O_3_-YAG):Ce eutectic samples of the different parts are demonstrated in Figure 2. The Al_2_O_3_ phase is visible as the dark stripes, and the YAG phase is visible as the light stripes of the images in Figure 2 (see also [24,25]). The obtained eutectic was grown under conditions close to equilibrium solidification. Still, its morphology looks like the “chines script”. As for similar eutectics produced at higher crystallization rates, which also applies to the (Al_2_O_3_-YAG):Ce eutectic system, they are far from stable [26]. Due to their closer physical properties (the refractive index and density of the sapphire Al_2_O_3_ (n = 1.77 and ρ = 3.99 g/cm^3^, respectively), they are a little smaller than the ones of YAG (n = 1.83 and ρ = 4.56 g/cm^3^) and the two phases may have less backscatter and total reflection loss at the edges. This leads to what is possible to create homogeneous light propagation without significant scattering losses at small refraction. Even for a thin sample, extending the optical path length results in more efficient Ce^3+^ excitation. As a result, the eutectic structures can produce light more effectively than conventional particle-dispersed LEDs [27].

**Figure 3:**
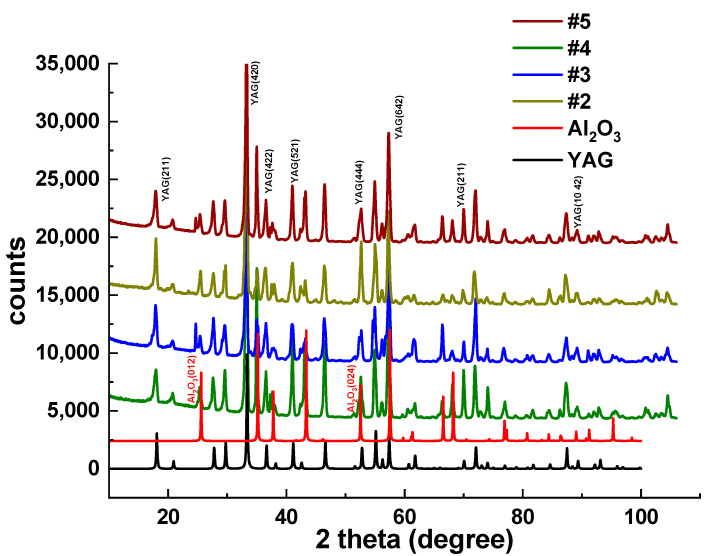
XRD patterns of 2–5 eutectic samples in comparison with Al_2_O_3_ (ICSD #63,647) and YAG (ICSD #23,848) phases.

For samples 2–5, the phase composition of eutectic crystals was identified. Figure 3 displays the XRD patterns of these two eutectic samples in comparison with the standard ICSD diffraction patterns for YAG (#23,848) and α-Al_2_O_3_ (corundum) (#63,647). The samples consist of pure YAG and corundum phase. The rate of melt solidification and the distance from the ingot seeding point determine the form and concentration of phases. The XRD patterns did not show (with an accuracy of the method of 0.1%) evidence of any other crystalline phases, particularly the yttrium aluminum perovskite YAlO_3_ (YAP) phase. Thus, the YAP phase does not dominantly appear under the solidification conditions utilized in this work. However, a small amount of YAP:Ce phase (below 0.1%) was detected in all the eutectic samples under study using the more sensitive PL investigation.

**Figure 4:**
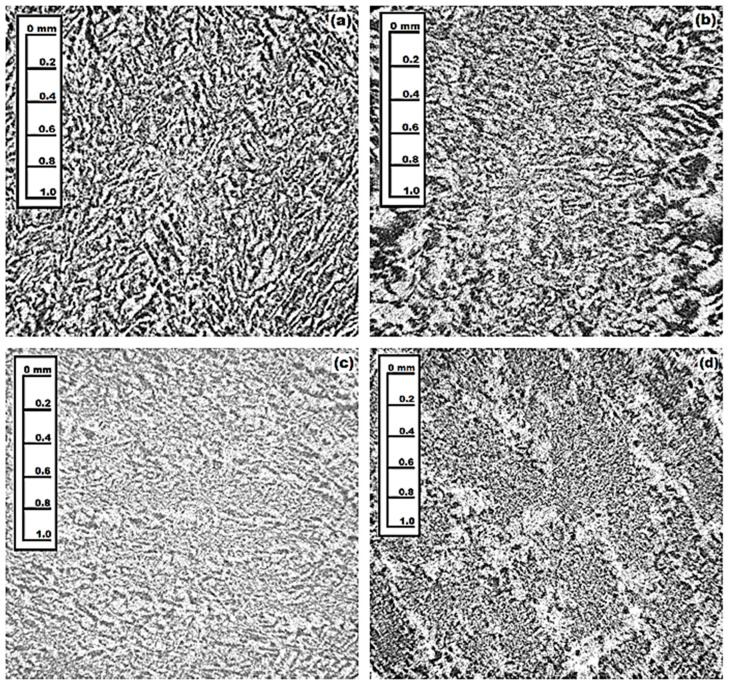
X-ray microtomography of the (Al_2_O_3_-YAG):Ce eutectic: garnet (white) and sapphire (gray) phases distribution for samples 2 (**a**), 3 (**b**), 4 (**c**), and 5 (**d**).

The X-ray microtomography (SkyScan 1272 spectrometer) investigations (Figure 4) of the samples under study show that the (Al_2_O_3_-YAG):Ce eutectic structure containing a random spatial distribution of the stripes of main garnet (white) and sapphire (gray) phases. The reconstructed 3D image of the eutectic structure is demonstrated in [28]. Table 2 shows the data obtained on the basis of the internal analysis of the images obtained by the microtomography (Figure 4). A detailed description of this method is described in the reference [29]. Namely, the analysis of the sample histogram, based on the mixture model, ref. [29] showed the volume fractions of both phases in the eutectic samples under study (Table 2).

**Figure 5:**
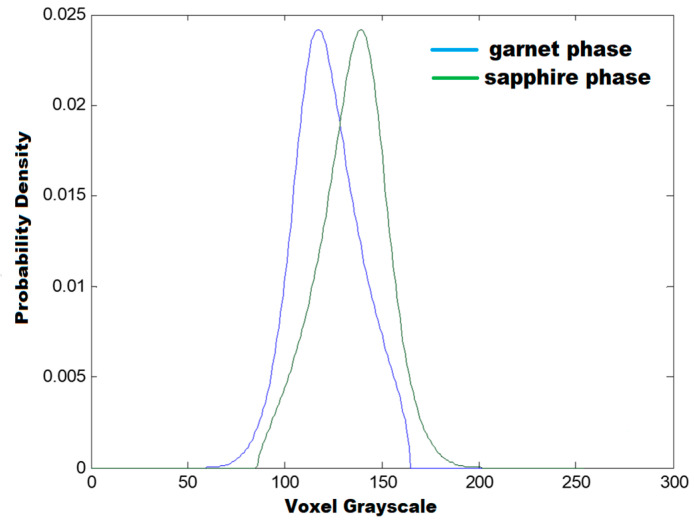
Graphs of the calculated density function in two subsets of voxels, corresponding to YAG (white) and sapphire (gray) phases in the microtomography image presented in Figure 4a for sample 2.

Figure 5 shows the determined graphs of gray level distributions of both phases in the image of sample two, proportional to its content (Table 2). However, it should be emphasized here that the difference in the position of both observed peaks is also related to the difference in the X-ray absorption ability of the YAG and sapphire phases μ~ρ *Z_eff_^4^, where ρ is the density and Z_eff_ is the effective atomic number, being equal to 35 for YAG and 11.2 for a sapphire.

## 4. Luminescent Properties

### 4.1. Catodoluminescence Spectra

**Figure 6:**
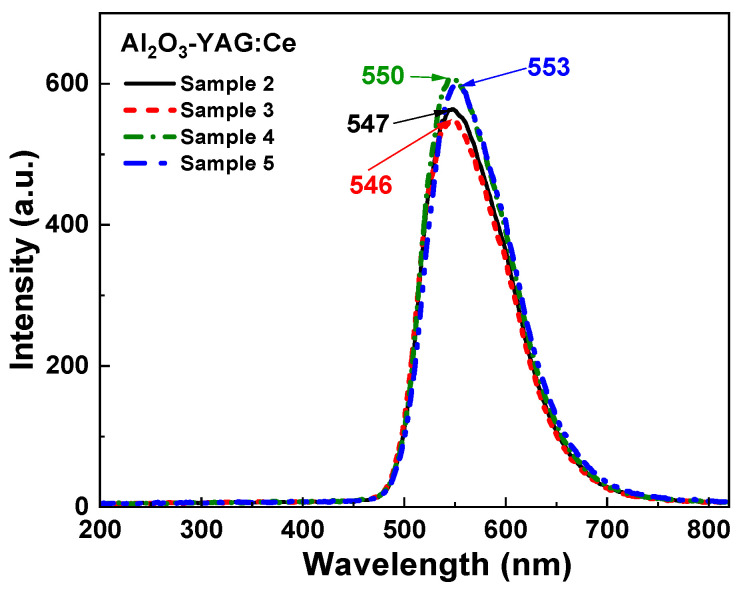
CL spectra of the (Al_2_O_3_-YAG):Ce eutectic samples under study.

The CL spectra of the samples under study (Figure 6) show only the intensive Ce^3+^ emission band peaked at the 547–553 nm range, caused by the presence of the YAG:Ce garnet phase. This result indicates also the existence of an effective energy transfer from the Al_2_O_3_:Ce sapphire phase to the YAG:Ce garnet phase in these eutectics under high-energy excitation. Interestingly, the maximum of the Ce^3+^ emission band in CL spectra is notably shifted in the red range with an increase in the crystallization rate and distance from the seed of eutectic (Table 1). This effect can be connected with a deviation of the Ce content in the respective eutectic samples and changing the crystal field strength in the dodecahedral positions of the garnet host [30] due to the incorporation of relatively large Ce^3+^ ions (ionic radius of 1.143 Ǻ in CN = 8) instead of Y^3+^ cations (1.019 Ǻ; CN = 8). Finally, that resulted in the observed long-wavelength shift of the Ce^3+^ emission spectra.

### 4.2. PL and PLE Spectra

**Figure 7:**
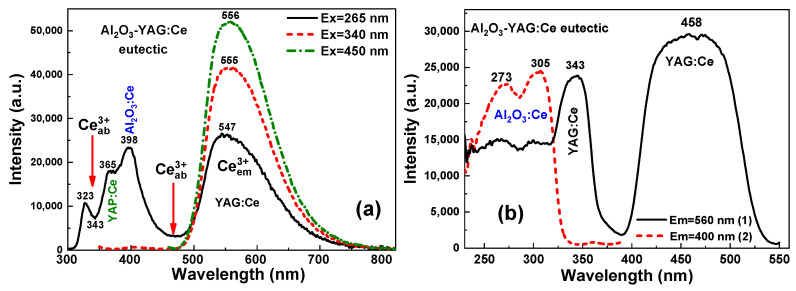
PL emission spectra (**a**) and PLE spectra (**b**) of (Al_2_O_3_-YAG):Ce eutectic (sample 4) under excitation at different wavelengths (**a**) and registration of Ce^3+^ luminescence in the garnet ((**b**), curve 1) and sapphire phase ((**b**), curve 2).

Figure 7 shows the PL and PLE spectra of the selected sample four of Ce^3+^ doped Al_2_O_3_-YAG eutectic. The radiation transitions of the Ce^3+^ ions in this sample were stimulated at 265, 340, and 450 nm light (Figure 7a). Such excitation results in a yellow-green PL emission band peaking at about the 547–556 nm range, corresponding to the 4f-5d transitions of Ce^3+^ ions in the YAG:Ce garnet phase. Namely, the spin-orbit splitting of the ground state allows for the decomposition of the PL emission band under 450 nm excitation into two components with centers at about 539 nm (5d^1^ → ^2^F_5/2_) and 584 nm (5d^1^ → ^2^F_7/2_) [31,32]. It can be assumed that the observed shift of the Ce^3+^ emission band is connected with the different probabilities of 5d^1^ → ^2^F_5/2,7/2_ transitions under different excitation wavelengths and peculiarities of the energy transfer between the sapphire and garnet phases.

The PLE spectra of the Ce^3+^ emission in the garnet phase in the (Al_2_O_3_-YAG):Ce eutectic sample four are displayed in Figure 7b, curve one. The two main bands in these spectra that peaked at 343 and 458 nm are related to the 4f-5d transitions of Ce^3+^ ions in the YAG:Ce garnet. The full width at a half maximum (FWHM) of the 458 nm excitation peak is about 100 nm, which suits well with blue LED chips of various emission wavelengths [33].

**Figure 8:**
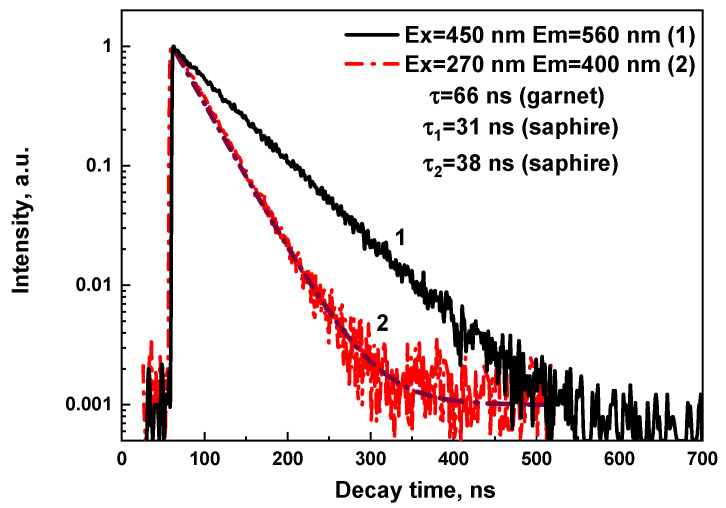
Decay kinetics of (Al_2_O_3_-YAG):Ce eutectic (sample 4) corresponding to the Ce^3+^ luminescence in the YAG:Ce (1) and Al_2_O_3_:Ce (2) phases under excitation in the respective PLE bands at 450 nm (1) and 270 nm (2).

The decay kinetics of the Ce^3+^ luminescence of (Al_2_O_3_-YAG):Ce eutectic, recorded in the vicinity of the Ce^3+^ emission band at 560 nm under excitation in the Ce^3+^ absorption band in the garnet phase at 460 nm, is shown in Figure 8, curve one. The decay kinetics of the Ce^3+^ luminescence in the garnet phase are strongly exponential and the decay constant is equal to 66 ns, which is characteristic of Ce^3+^ luminescence in other garnet compounds [34].

The PL emission spectra of this (Al_2_O_3_-YAG):Ce eutectic sample four also show the complex emission band in the UV range, peaked at 396 nm (Figure 7a, curve one), corresponding to the Ce^3+^ luminescence in the Al_2_O_3_:Ce phase [34]. The bands peaked at 273 and 305 nm in the PLE spectrum of this luminescence monitored at 400 nm (Figure 7b, curve 2), which are caused by the respective 4f–5d (^2^E and ^3^T_2g_) transitions of Ce^3+^ ions in the Al_2_O_3_ host [35]. Furthermore, the effective energy transfer processes are observed between Ce^3+^ ions in the Al_2_O_3_ phase to the garnet phase under high-energy excitation and excitation in the corresponding UV bands. The confirmation of such transfer is also quite nonexponential decay kinetics of the Ce^3+^ luminescence in the Al_2_O_3_:Ce phase under excitation at 260 nm in the vicinity of the respective PLE band (Figure 8, curve two). The approximation of the respective decay curve two in Figure 8 shows the presence of the two components with decay times of t_1_ = 31 ns and t_2_ = 38 ns, respectively. Furthermore, the 35 ns average decay time was used for the characterization of the decay profiles of this luminescence and its value is noticeably lower, though consistent with the lifetime of the Ce^3+^ luminescence in the Al_2_O_3_:Ce single crystalline films, being equal to 42 ns [35]. Moreover, such nonexponential decay profiles of the Ce^3+^ luminescence in the sapphire phase in the eutectic samples are strongly temperature dependent. This means that the energy transfer from the sapphire phase to the garnet phase increases with increasing temperatures. These new results will be presented soon in a separate paper.

It is worth noting here that the presence of the small content (less than 1%) YAP:Ce perovskite phase was also tested in the PL emission spectra of the (Al_2_O_3_-YAG):Ce eutectic sample under 265 nm excitation (Figure 6, curve 1). Most probably, the observed bump peaked at 365 nm in this spectrum in Figure 7a corresponds to the Ce^3+^ luminescence in the YAP host. However, the shape of the Ce^3+^ emission band in the YAP:Ce phase is strongly affected by the presence of the Ce^3+^ absorption band in the garnet phase, which peaked at 343 nm.

## 5. WLED Prototype Creation

**Figure 9:**
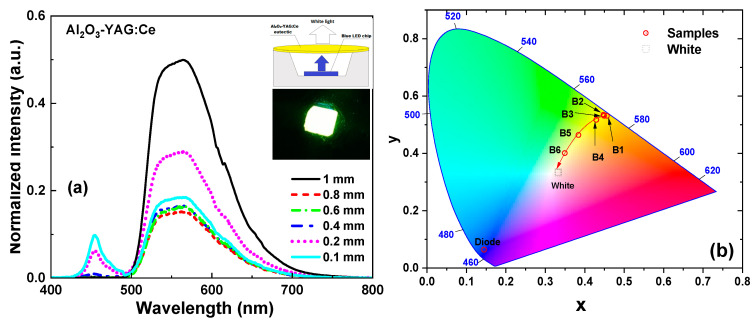
The spectral performance of (Al_2_O_3_-YAG):Ce eutectic samples under 450 nm blue LED excitation (**a**); color coordinates of (Al_2_O_3_-YAG):Ce eutectic based LEDs in CIE-1931 color space chromaticity diagram (**b**).

We have also tested the photoconversion (PC) prototypes of WLEDs, prepared using a blue 450 nm LED chip and Ce-doped Al_2_O_3_-YAG eutectic photoconverters (pc) with thicknesses in the 0.15–1 mm range (Figure 9). The results of the tests are quite encouraging. The emission spectrum for these WLEDs covers the visible range from 460 to 820 nm with warmer light, in comparison with the standard YAG:Ce photoconverter [36]. Figure 9 shows also the dependence of the PC properties of the (Al_2_O_3_-YAG):Ce eutectic on the thickness of the samples. Namely, when the eutectic thickness grows, the blue LED’s intensity declines while the intensity of the yellow emission band rises. The blue light is almost entirely absorbed, and the yellow emission achieves its peak intensity at a thickness of around 1.0 mm.

The CIE-1931 chromaticity diagram in Figure 8b also displays the changes in color coordinates (x, y) of the (Al_2_O_3_-YAG):Ce eutectic with different thicknesses in the 0.15–1 mm range. The coordinates have a nonlinear distribution, and the x and y values rise with the eutectic thickness. We assumed that decreasing the thicknesses from 0.4 mm to 0.1 mm can be used to create white colors with various color temperatures. The CIE chromaticity coordinates of WLED prototypes are shown in Table 3. Namely, the combination of the (Al_2_O_3_-YAG):Ce eutectic sample with different thicknesses in the 0.4–0.15 mm range enables tuning of the white light shades from warm white (CCT = 3810 K) to warm/daylight white (CCT~5120 K) [37,38]. Based on the obtained results, presented in Figure 9 and Table 3, we can expect that the ideal white color can be achieved for a eutectic convertor thickness of about 0.1–0.15 mm under 450 nm LED excitation. Due to significantly smaller lightguide losses and the small intrinsic reflection losses, the thickness of the eutectic converter is significantly smaller than the optimal thickness of the YAG:Ce (0.25%) crystal counterpart (0.5–0.55 mm), grown from the melt with nominal ceriun content of 1 molar % [39].

We have provided the results of measurements of the luminous efficiency (Lm/W) of the WLED prototypes with a gradual reduction of the eutectic thicknesses from 1 to 0.15 mm (the last column in Table 3). As can be seen from this table, the LE value steadily increases when the thickness of the eutectic converter decreases up to 0.15 mm. Finally, the WLED prototype eutectic converter with a thickness of 0.15 mm shows a luminous efficiency of above 140 Lm/W.

## 6. Conclusions

In this study, Ce^3+^ doped Al_2_O_3_-YAG eutectic samples were crystallized using the horizontal directional crystallization method at a growth temperature of 1835 °C, with varying crystallization rates in the 1–7.5 mm/h range. The microstructure of the eutectic samples was analyzed using scanning electron microscopy, X-ray diffraction, and X-ray microtomography. The samples consisted primarily of the YAG and Al_2_O_3_ phases, with a small amount (below 1%) of the YAP:Ce perovskite phase also observed in Al_2_O_3_-YAG via a photoluminescence spectra. The eutectic morphology exhibited a stripe-like channel structure with the random distribution of the garnet and sapphire phases. The content of these phases in the stripes varied depending on the growth rate of the eutectic samples, with ranges of 52–54% and 48–46% for the garnet and sapphire, respectively.

The luminescent properties of the eutectic samples demonstrated the dominant Ce^3+^ emission band in the garnet phase and the weak Ce^3+^ luminescence in the sapphire phase. Furthermore, the effective energy-transfer processes between Ce^3+^ ions in the Al_2_O_3_ and YAG garnet phases were observed under high-energy excitation, as well as excitation in the UV Ce^3+^ absorption bands of sapphire.

We have investigated the photoconversion properties of (Al_2_O_3_-YAG):Ce eutectic samples with different thicknesses, under excitation by a blue LED. We also tested prototypes of white LEDs prepared using (Al_2_O_3_-YAG):Ce eutectic photoconverters, with thicknesses ranging from 0.15 to 1 mm, and a blue 450 nm emitting LED chip. We have found that the combination of (Al_2_O_3_-YAG):Ce eutectic with thicknesses in the 0.4–0.15 mm range and 450 nm LED excitation enables tuning of the white light tones from warm white (CCT ~ 3800 K) to white daylight (CCT ~ 5100 K). Furthermore, the ideal white color can be achieved for a eutectic converter thickness in the 0.1–0.15 mm range, and the respective WLED prototype exhibits a luminous efficiency above 140 Lm/W.

## Figures and Tables

**Table 1:** Modes of crystallization of eutectic samples.

Sample	Rate of Crystallization, mm/h	Melt Temperature, °C	Gradient, K/cm	Al_2_O_3_, Surf. %	YAG, Surf. %
2	1.6	1835	30	47.1 ± 2.4	52.9 ± 2.4
3	1	1835	30	46.1 ± 1.3	54.0 ± 1.3
4	2.8	1835	30	44.7 ± 1.3	55.3 ± 0.7
5	7.5	1835	30	45.2 ± 0.7	54.8 ± 0.7

**Table 2:** The sapphire and garnet phase proportions of the eutectic samples.

Sample	Al_2_O_3_ Phase Content, %	YAG Phase Content, %
2	54.39	46.61
3	50.02	49.98
4	45.58	54.42
5	46.60	53.4

**Table 3:** CIE chromaticity coordinates, CTT and luminous efficiency of a WLED lamp fabricated on the base of 450 nm LED chip and (Al_2_O_3_-YAG): Ce eutectic (sample 4) with different thicknesses.

Thicknesses of Sample 4 h, mm	CIE Coordinates		CCT, K	CRI	**LE (lm/W)**
	x	y			
1 (B1)	0.4567	0.5299	3489	39.7	68
0.8 (B2)	0.4498	0.5306	3580	46.1	81
0.6 (B3)	0.4483	0.5341	3620	42.3	107
0.4 (B4)	0.4300	0.5165	3810	55.9	120.4
0.2 (B5)	0.4047	0.4633	4530	67.4	132
0.15 (B6)	0.35	0.40	5120	72.5	142.5

## Data Availability

Not applicable.
